# Coagulation Status Assessment in Dogs with Chronic Enteropathy Using Viscoelastic Point-of-Care Coagulation Monitor

**DOI:** 10.3390/ani15111571

**Published:** 2025-05-28

**Authors:** María José Marín Lucas, Tim Sparks, Chantal Rosa

**Affiliations:** 1 Northwest Veterinary Specialists, Linnaeus Veterinary Ltd., Delamere House, Ashville Point, Beechwood, Sutton Weaver, Runcorn WA7 3FW, UK; maria.marin@scarsdalevets.com; 2 Waltham Petcare Science Institute, Melton Mowbray LE14 4RT, UK; tim.sparks@effem.com

**Keywords:** canine, chronic inflammatory enteropathy, hypercoagulability, viscoelastic testing, VCM Vet^®^, protein-losing enteropathy, canine chronic enteropathy clinical activity index, immunosuppressive therapy

## Abstract

Canine chronic inflammatory enteropathy (CIE) has been associated with coagulation abnormalities, predisposing affected dogs to a hypercoagulable state and potential thromboembolic events. This study aimed to evaluate the coagulation status in dogs with CIE using a viscoelastic point-of-care device, a Viscoelastic Coagulation Monitor (VCM Vet^®^). A retrospective review of medical records identified 38 dogs diagnosed with CIE that underwent VCM Vet^®^ testing. Coagulation profiles were classified as hypercoagulable, normocoagulable, or hypocoagulable. The results demonstrate that 81.5% of dogs exhibited hypercoagulability, and significant associations were found between the coagulation status and the type of CIE. Hypercoagulability was more commonly found in immunosuppressive-responsive enteropathy (IRE) cases. Albumin and cobalamin were significantly higher in food-responsive enteropathy, whereas the canine chronic enteropathy clinical activity index (CCECAI) was significantly higher in immunosuppressive-responsive enteropathy and non-responsive enteropathy. One dog with protein-losing enteropathy (PLE) was suspected of having developed possible pulmonary thromboembolism. These findings reinforce previous reports of hypercoagulability in CIE and suggest that VCM Vet^®^ is a valuable and easy tool to assess coagulation abnormalities in a clinical setting. Further investigation is warranted to evaluate the clinical implications of hypercoagulability in CIE and the potential role of anticoagulant therapy in disease management.

## Simple Summary

This study investigates the coagulation status of dogs with chronic inflammatory enteropathy (CIE) using a portable device called a Viscoelastic Coagulation Monitor (VCM Vet^®^). The aim was to determine whether these dogs show an abnormal coagulation status, as seen in previous studies using other coagulation methods, such as thromboelastography and thromboelastometry. Dogs were classified as having immunosuppressive responsive enteropathy (IRE), non-responsive enteropathy (NRE), food-responsive enteropathy (FRE), or microbiota-related modulation-responsive enteropathy (MrMRE) depending on their clinical response to treatment. The results show that most dogs with CIE were hypercoagulable, particularly the IRE group. Albumin, cobalamin, and disease severity scores, assessed using the canine chronic enteropathy clinical activity index (CCECAI), were significantly different in dogs receiving immunosuppressive medication. Thromboembolic events were recorded when present. The findings highlight the potential risks of abnormal hemostasis in dogs with CIE and suggest that the VCM Vet^®^ could be a useful tool in veterinary medicine with the advantage of being a hand-held and easy-to-use device.

## 1. Introduction

The Viscoelastic Coagulation Monitor Vet (VCM Vet^®^, Entegrion, Durham, NC 27703, USA) is a portable advanced viscoelastic testing device that provides real-time rapid hemostasis assessment, including clot formation, retraction, and lysis. It also measures the rate of clot formation, clot strength, and stability on a small volume of a whole blood sample [1,2]. The acronym VCM refers to Viscoelastic Coagulation Monitor device (VCM Vet^®^) and will be used throughout the manuscript. Reference intervals for the VCM variables have been established for both dogs and cats [3,4,5]. A recent study comparing the VCM with thromboelastography (TEG) in dogs with suspected hemostatic disorders found a variable correlation in clot formation parameters, including variable lysis parameter agreement between these two devices, but strong correlation in the final cloth strength [4].

Coagulation assessment is crucial in various diseases, including canine chronic inflammatory enteropathies (CIEs) [6,7]. Coagulation status assessed using the VCM has also been found in dogs with pulmonary hypertension [8], primary immune-mediated hemolytic anemia [9], healthy dogs during the perianesthetic period [10], healthy dogs after central catheter placement [11], and healthy Greyhounds and non-Greyhound blood donor dogs [12]. Additionally, VCM coagulation profiles have been compared with TEG and thromboelastometry (ROTEM) in both healthy and diseased canine patients [4,5,13]. Feline coagulation status assessed using the VCM has also been investigated [14,15,16,17,18].

Canine CIE is characterized by persistent or recurring gastrointestinal signs (≥3 weeks) such as vomiting, diarrhea, nausea, borborygmi, loss of appetite, flatulence, abdominal pain, and/or weight loss [19,20,21,22]. To diagnose CIE, extra-gastrointestinal diseases such as renal, pancreatic, or hepatic diseases; hypoadrenocorticism; hypercalcemia; infectious diseases; or neoplasia must be excluded [23,24]. Once diagnosed, CIE is subclassified based on the clinical response to treatment into (1) food-responsive enteropathy (FRE); (2) microbiota-related modulation-responsive enteropathy (MrMRE); (3) immunosuppressant-responsive enteropathy (IRE); and (4) non-responsive or refractory enteropathy (NRE) [19,20,21].

Additionally, protein-losing enteropathy (PLE) is a syndrome characterized by protein loss across the intestinal wall that can be present in CIE, leading to hypoalbuminemia [25,26,27]. An assessment of coagulation in canine CIE with [6,7,28,29] and without PLE [7,29] has previously been reported using standard TEG [6,7,29] and ROTEM [28], but also other hand-held devices such as TEG® 6 s (Haemonetics® Corporation, Braintree, MA, USA) [30], generally indicating a hypercoagulable state. This hypercoagulability in canine CIE may be associated with albumin and antithrombin (AT) loss, although one study suggested that hypercoagulability was not correlated with the severity of decrease in these parameters [6]. Other proposed mechanisms for hypercoagulability include the administration of prednisolone [31,32] or low-grade gastrointestinal bleeding, which might lead to anemia and/or thrombocytosis, thereby altering clot dynamics [15,32]. A study assessing dogs with CIE and normal serum albumin were found hypercoagulable as measured using TEG [7], possibly due to the hyperfibrinogenemia that occurs in CIE [6,7,33].

The primary aim of our study was to describe the coagulation status of dogs based on their type of CIE using the VCM and assess whether our results are in accordance with previous findings in canine CIE described by using TEG, ROTEM, and TEG^®^ 6 s. Our hypothesis is that dogs diagnosed with CIE would be hypercoagulable when assessed using VCM irrespective of the clinicopathologic variables, as previously reported using other methods/equipment. Our second aim was to compare the coagulation status (hypercoagulable, normocoagulable, hypocoagulable, or hyperfibrinolytic), the VCM variables (CT, CFT, α angle, A10, A20, and MCF), and the type of CIE (FRE, MrMRE, IRE, and NRE) with clinical and clinicopathological variables. A third aim was to evaluate whether any patients experienced thromboembolic events and whether coagulation status could predict such occurrences, given that previous studies have reported a moderate risk of thromboembolism in patients with PLE [34].

To date, as far as the authors are aware, no studies have specifically evaluated the coagulation status in dogs based on the type of CIE using the VCM specifically, a portable point-of-care device that has been increasingly used to characterize hemostatic abnormalities in veterinary medicine. This equipment has the advantage of being a cartridge-based system, which does not require manual pipetting and provides rapid results. This study is valuable in assessing the clinical utility of VCM, a tool that is becoming more widely available in veterinary practice.

## 2. Materials and Methods

### 2.1. Study Population

Medical records from client-owned dogs referred to a specialist referral center in the United Kingdom from September 2021 to February 2025 were retrospectively reviewed to identify dogs with CIE that underwent VCM testing. Ethical approval for this study was obtained from the Royal College of Veterinary Surgeons ethical committee.

Dogs were included if they presented with chronic (≥3 weeks) gastrointestinal signs and had a strong suspicion or confirmed diagnosis of CIE. Included dogs were assessed based on hematology; serum biochemistry; serum cortisol; folate and cobalamin concentrations; fecal analysis, including *Giardia, Salmonella*, and *Campylobacter*; abdominal ultrasound; and/or Computed Tomography. Bile acids or a bile acid stimulation test was performed in dogs with hypoalbuminemia and suspected liver dysfunction based on bloodwork and/or abdominal imaging. Urinalysis and urine protein-to-creatinine (UPC) ratio was performed in all hypoalbuminemic patients to exclude protein-losing nephropathies.

Hypoadrenocorticism was excluded in all cases if the basal cortisol level was >55 nmol/L or if the results of the adrenocorticotropic hormone stimulation test were within normal limits [35]. Trypsin-like-immunoreactivity (TLI) was performed in dogs with suspicion of exocrine pancreatic insufficiency (weight loss, polyphagia, and/or diarrhea), and normal results were found in all performed cases.

Patients were excluded if they were diagnosed with diseases other than CIE or neoplasia, had received corticosteroids or non-steroidal anti-inflammatory drugs (NSAIDs) within a week of VCM testing, or if their medical records were incomplete. A total of 624 VCM tracings were reviewed. From these, 115 patients were diagnosed with a gastrointestinal disease. Seventy-seven dogs were excluded due to a diagnosis of gastrointestinal foreign bodies, infectious diseases (Parvovirus or *Giardia*), or gastrointestinal ulceration from NSAID administration, as well as dogs diagnosed with CIE already on prednisolone treatment and those with pancreatitis, acute gastroenteritis, septic peritonitis, neoplasia, intussusception, oronasal fistula, non-associative immune-mediated thrombocytopenia, and cholangiohepatitis. Thirty-eight dogs met the inclusion criteria and were retrospectively included.

Signalment (age, sex and neuter status, and breed), type and duration of clinical signs were extracted from the medical records. Mean age was 7 years (range 6 months–13 years), and there were 21 female dogs (neutered, *n* = 18; entire, *n* = 3) and 17 male dogs (neutered, *n* = 8; entire, *n* = 9). Breeds included crossbreeds (*n* = 10), Labrador Retriever (*n* = 4), Dachshund (*n* = 3), Chihuahua (*n* = 2), Cocker Spaniel (*n* = 2), and one of each of German Shepherd, Pointer, Jack Russell Terrier, West Highland White Terrier, Alaskan Malamute, New Zealand Huntaway, Hungarian Vizsla, English Bull Terrier, French Bulldog, Cavalier King Charles Spaniel, Rough Collie, Pomeranian, Pug, Bichon Frise, Border Collie, Yorkshire Terrier and Hungarian Puli.

Mean duration of clinical signs before presentation was 12 weeks (range 3–60 weeks) and included vomiting (*n* = 28), diarrhea (*n* = 27), reduced appetite (*n* = 18), weight loss (*n* = 11), hematochezia (*n* = 11), lethargy (*n* = 8), borborygmi (*n* = 7), abdominal discomfort (*n* = 6), polyuria/polydipsia (*n* = 4), regurgitation (*n* = 3), melena (*n* = 2), ascites/abdominal distension (*n* = 2), hypersalivation (*n* = 1), peripheral edema (*n* = 1), and flatulence (*n* = 1). Clinical signs are summarized in Table 1.

### 2.2. Laboratory Testing

Blood was collected from the jugular vein in all patients as none were clinically suspected to have thrombocytopenia at the time of sampling. Jugular venipuncture was preferred to minimize the risk of platelet clumping and ensure optimal sample quality. A 22 G needle and syringe were used for blood sampling, and approximately 4–5 mL was required for all determinations, including 0.5 mL in EDTA for hematology, 0.35 mL whole blood for VCM analysis, and 3–3.5 mL in serum tubes for biochemistry. These blood samples were used to perform hematology, serum biochemistry, cobalamin, folate, TLI, bile acids, cortisol, and VCM analyses.

Hematology was analyzed using an automated hematology analyzer (Sysmex XT-2000 iV, Sysmex Corporation, Kobe, Japan). Serum biochemistry, cobalamin, folate, TLI, bile acids, and cortisol were measured using blood samples submitted to the Veterinary Pathology Laboratory in the United Kingdom (VPG, UK). All tests were performed following the laboratory’s standard operating procedures. Serum biochemistry was analyzed using the IDEXX Catalyst DX (IDEXX Laboratories, Inc., Westbrook, ME, USA). Cobalamin, folate, and TLI were measured using the IDEXX Cobalamin/Folate/TLI assay (IDEXX Laboratories, Inc., Westbrook, ME, USA). Bile acids were assessed using the IDEXX Bile Acids test (IDEXX Laboratories, Inc., Westbrook, ME, USA). Cortisol levels were analyzed using IMMULITE 2000 (Siemens Healthineers, Erlangen, Germany). Reference intervals were provided by the Veterinary Pathology Laboratory based on species-specific validation for canine patients.

Hematocrit (HCT), platelet count, albumin, cobalamin, and folate were recorded. They were considered normal if the values were within the laboratory reference intervals (HCT, 37–55%; platelet count, 140–700 × 10^9^/L; albumin, 24–40 g/L; cobalamin, 400–908 ng/L [36,37,38]; and folate, 7.7–24.4 ng/L). 

Anemia was classified as mild (HCT 30–36%), moderate (HCT 20–29%), or severe (HCT < 20%) based on the degree of the drop in HCT. All dogs with a platelet count below the reference range used by the laboratory (140–700 × 10^9^/L) on the analyzer had a manual blood film examination to confirm a normal platelet count by performing average platelet count in 10 oil immersion fields and multiplying by 15,000 [39].

Hypoalbuminemia was defined as a serum albumin concentration of <24 g/L, while concentrations ≥ 24 g/L were considered consistent with normoalbuminemia. Hypoalbuminemia was stratified into the following groups: mild (20–24 g/L), moderate (15–19 g/L), and severe (<15 g/L). Hypocobalaminemia was classified as mild (200–400 ng/L), moderate (100–199 ng/L), or severe (<100 ng/L). Hypercobalaminemia was defined as serum cobalamin > 908 ng/L [38].

Endoscopic gastrointestinal biopsies were performed, and histological results were recorded in 19 patients.

### 2.3. Coagulation Testing

For the VCM analysis, a volume of 350 µL of whole blood was transferred as soon as collected into a pre-heated (37 °C) VCM cartridge, and analysis was started immediately, according to the manufacturer’s instructions [1], by laboratory technicians who had been previously trained on how to use the equipment. Variables measured using the VCM are similar to the ROTEM and TEG instrumentation and include the clot time (CT), clot formation time (CFT), α-angle, maximum clot firmness (MCF), A10 (amplitude at 10 min), A20 (amplitude at 20 min), and lysis indices at 30 min (LI30) and 45 min (LI45) after CT. 

Using the laboratory reference intervals, VCM tests for included dogs were categorized as hypocoagulable, hypercoagulable, or normocoagulable [5]. Hypocoagulability was defined as the presence of ≥2 parameters indicating this state, including prolonged CT/CFT, decreased α angle, decreased A10/A20, and/or decreased MCF. Hypercoagulability was defined as the presence of ≥2 parameters indicating this state, such as shortened CT/CFT, increased α angle, increased A10/A20, and/or increased MCF [40]. Inconclusive tracings were defined as those with both hypocoagulable and hypercoagulable VCM parameters. Hyperfibrinolysis was defined as a decreased LI30 or LI45 value compared to the reference intervals established for this equipment. The VCM tracings were classified as normal if they were not hypercoagulable, hypocoagulable, inconclusive, or hyperfibrinolytic [13,41].

### 2.4. Disease Categorization

Dogs were classified as having FRE if there was a positive response to one or more dietary trials, and the response was deemed positive if there was an improvement in clinical signs and CCECAI scores. Among the 16 patients included in the FRE group, 15 responded to the first diet trial, 12 of them responded to a hydrolyzed protein diet, and 3 of them responded to a low-fat diet (2 to a gastrointestinal low-fat diet and 1 to a home-cooked low fat diet), while 1 patient required 2 diet trials (first a hydrolyzed diet and then a novel protein diet) to resolve the clinical signs. 

One dog underwent several diet trials with no clinical response but improved after the addition of a high-dose multi-strand probiotic and was therefore classified as having MrMRE. 

Dogs were classified as having IRE if diet and microbiota-related modulation treatment trials failed, and immunosuppressive medication was necessary to control the clinical signs (all of them had prednisolone at a dose of 1–2 mg/kg orally once daily, 3 of them required the addition of a second immunosuppressant—cyclosporin was used in 2 dogs at a dose of 5–10 mg/kg orally once daily, and chlorambucil in one dog dosed at 2 mg/m^2^ orally once daily). Seven out of fifteen patients with PLE were prescribed antithrombotic prophylactic therapy with clopidogrel (loading dose of 4–10 mg/kg once on the first day of therapy followed by 2 mg/kg once daily as a maintenance dose). All these medications were initiated after VCM testing was performed.

Dogs were classified as having NRE when no clinical response was seen despite diet, probiotics, and/or fiber and immunosuppressive therapy. 

Canine chronic enteropathy clinical activity index (CCECAI) scores were calculated, and severity of disease was defined as insignificant (0–3), mild (4–5), moderate (6–8), severe (9–11), or very severe (≥12) [42,43].

### 2.5. Statistical Analysis

Because of the categorical or skew nature of the data, non-parametric methods were used throughout. Categorical data were summarized as frequencies and percentages and compared between groups using Fisher’s exact test. Continuous/ordinal data were summarized by medians and absolute ranges and compared between the groups using Mann–Whitney or Kruskal–Wallis tests adjusted for ties. The MrMRE group was excluded from the latter because it was only represented by a single dog. Associations between continuous/ordinal variables were tested using Spearman rank correlations. Significance was taken as *p* < 0.05. Analysis was conducted in Minitab21 and R 4.3.3. Sample size calculations were also conducted using a significance level of 0.05 and a power of 80%; a sample of 33 dogs was required to detect a correlation coefficient of r_s_ = 0.6, which indicates moderately strong correlations, using a non-parametric test.

Groups were defined as hypercoagulable, normocoagulable or hypocoagulable according to the above Section 2.3. Differences between the coagulation status groups and between type of CIE for clinicopathological variables (age, duration of clinical signs, HCT, platelet count, albumin, cobalamin, folate and CCECAI scores) were assessed by Kruskal-Wallis tests adjusted for ties and for sex/neuter status using Fisher exact tests. The VCM variables (CT, CFT, MCF, α angle, A10 and A20) were tested for association with clinicopathological variables using Spearman rank correlation and for differences between types of CIE using Kruskal-Wallis tests adjusted for ties.

## 3. Results

### 3.1. Group Characteristics

A total of 38 dogs were included. Of these, 31 dogs were hypercoagulable, 7 dogs were normocoagulable, and no dog was found hypocoagulable or hyperfibrinolytic. All included VCM results and variables are summarized in Table 2. The most frequent abnormalities observed were increased A20 in 37 dogs, elevated MCF in 29 dogs, and increased A10 in 26 dogs, followed by increased α angle in 13 dogs, decreased CFT in 10 dogs, and decreased CT in 6 dogs.

Of the 38 dogs, 16 were diagnosed with IRE, 16 with FRE, 1 with MrMRE, and 5 with NRE. Clinical and clinicopathological data, including age, sex and neuter status, duration of clinical signs, HCT, platelet count, albumin, cobalamin, folate, CCECAI score, VCM result, and type of CIE, are summarized in Table 3, and abnormal values are marked with an asterisk (*).

Moderate non-regenerative anemia was observed in one dog (case 22, Table 3), likely secondary to chronic disease (FRE-PLE), which was resolved following the resolution of clinical signs. Another dog exhibited severely regenerative anemia (case 14, Table 3) with a hematocrit of 10% secondary to gastrointestinal blood loss documented by the presence of melena and hematochezia. Mild thrombocytopenia was identified in one patient (case 19, Table 3), but a manual blood smear confirmed that the thrombocytopenia was artifactual due to platelet clumping.

Hypoalbuminemia was observed in 15 dogs, with mild hypoalbuminemia (20–24 g/L) being observed in 2 cases, moderate hypoalbuminemia (15–19 g/L) in 7 dogs, and severe hypoalbuminemia (<15 g/L) in 6 dogs [44]. Clinical and clinicopathological values were summarized by minimum and maximum values based on the type of CIE, as shown in Table 4, organized by the type and subtype of CIE.

Hypocobalaminemia was detected in 21 cases, of which 11 were mild (200–400 ng/L), 9 were moderate (100–199 ng/L), and 1 was severe (<100 ng/L). Hypercobalaminemia (>908 ng/L) [38] was detected in six patients. Hypofolatemia was detected in nine cases, and hyperfolatemia was found in one dog. Cobalamin and folate were not measured in one patient that was receiving oral supplementation when seen, and no prior blood assessment was performed by the referring veterinarian.

Histology was performed by board-certified pathologists and/or residents in 19 of the 38 included patients. Histology from the stomach (*n* = 19), duodenum (*n* = 19), and/or colon (*n* = 8) were consistent with an inflammatory enteropathy that was classified as neutrophilic, lymphocytic, plasmocytic, eosinophilic, or a combination of these. Inflammation was further classified as mild, moderate, or severe. Lymphangiectasia was found in two patients with IRE, lamina propria fibrosis in three IRE cases, crypt abscessation in two IRE cases, and villous atrophy in one IRE case. Of the 19 dogs without histology results, 12 were classified as having FRE and 1 as MrMRE, all of which had a resolution of the clinical signs after one or more diet trials and/or the addition of probiotics. These FRE dogs remained well controlled during the available follow-up period. Four dogs were classified as having IRE and did not undergo endoscopy and biopsies due to financial restraints but responded to immunosuppressive therapy, and the remaining two dogs were classified as having NRE and died before endoscopy could be performed without any response to previous treatments prescribed.

### 3.2. Coagulation Status (Normocoagulable vs. Hypercoagulable) Association with Type of CIE

In the IRE group, all patients were hypercoagulable, while in the FRE group, 75% were hypercoagulable and 25% normocoagulable. The MrMRE case was normocoagulable. In the NRE group, 60% of dogs were hypercoagulable, and 40% were normocoagulable (Table 5).

**Figure 1:**
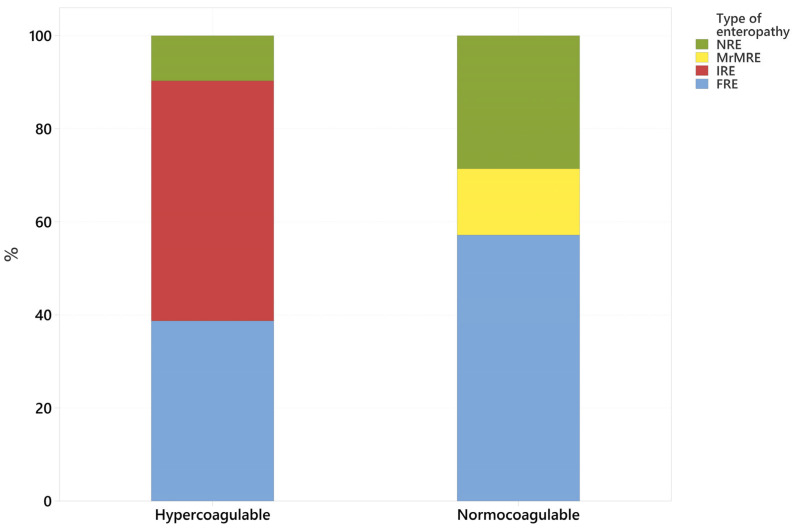
Distribution of type of CIE by coagulation status. Abbreviations: FRE = food-responsive enteropathy; IRE = immunosuppressant-responsive enteropathy; MrMRE = microbiota-related modulation-responsive enteropathy; NRE = non-responsive enteropathy.

Overall, patients diagnosed with suspected CIE in our hospital displayed a hypercoagulable trace (81.5%, *n* = 31), with 18.5% (*n* = 7) having a normocoagulable trace (Figure 1). No hypocoagulable or hyperfibrinolytic results were obtained.

From the 31 hypercoagulable results, 16 patients were classified as having IRE (51.6%), 12 were classified as having FRE (38.7%), and 3 were classified as having NRE (9.6%). Of the seven normocoagulable patients, four were classified as having FRE (57%), two were classified as having NRE (28%), one was classified as having MrMRE (14.2%), and none were classified as having IRE (Figure 1 and Table 4).

Fisher’s exact test (*p* = 0.012) indicated a statistically significant difference between the coagulation status and the type of CIE, with hypercoagulability more commonly observed in dogs with IRE and normocoagulability more commonly observed in dogs with FRE.

### 3.3. Clinical and Clinicopathological Variables Compared with Coagulation Status and Type of CIE

Coagulation status was not significantly associated with age (*p* = 0.763), sex and neuter status (*p* = 0.804), duration of clinical signs (*p* = 0.427), HCT (*p* = 0.970), platelet count (*p* = 0.707), albumin (*p* = 0.219), cobalamin (*p* = 0.712), folate (*p* = 0.877), or CCECAI score (*p* = 0.296).

**Figure 2:**
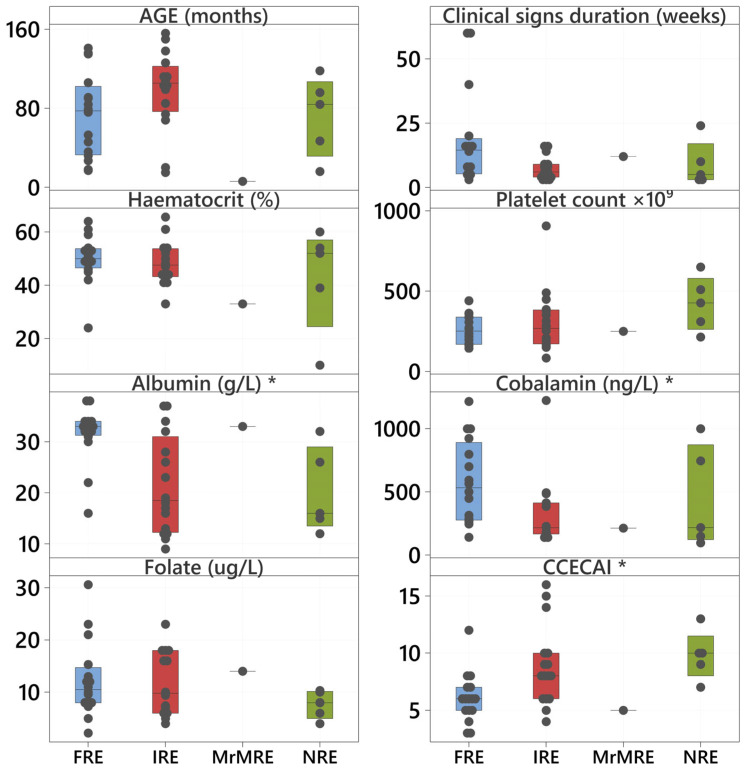
Boxplots of clinicopathological variables by type of CIE (blue boxplots represent FRE; red boxplots represent IRE; and green represents NRE). The boxes represent the median and interquartile range; individual values are indicated by solid symbols. The three variables indicated by an asterisk differ significantly between types of CIE according to Kruskal-Wallis tests adjusted for ties. Abbreviations: CCECAI = canine chronic enteropathy clinical activity index; FRE = food-responsive enteropathy; IRE = immunosuppressant-responsive enteropathy; MrMRE = microbiota-related modulation-responsive enteropathy; NRE = non-responsive enteropathy.

The type of CIE was not significantly associated with age (*p* = 0.164), sex and neuter status (*p* = 0.225), duration of clinical signs (*p* = 0.079), HCT (*p* = 0.679), platelet count (*p* = 0.139), or folate (*p* = 0.420) (Figure 2).

Serum albumin (*p* = 0.005), the cobalamin concentration (*p* = 0.028), and CCECAI scores (*p* = 0.004) were significantly different between the types of CIE (Figure 2 marked with asterisks, Table 6). Albumin and cobalamin concentrations were higher in dogs with FRE vs. dogs with IRE and NRE. The score severity index, CCECAI, was higher in dogs with IRE and NRE when compared with FRE cases.

### 3.4. VCM Variables: Association with Clinical and Clinicopathologic Variables and Type of CIE

**Figure 3:**
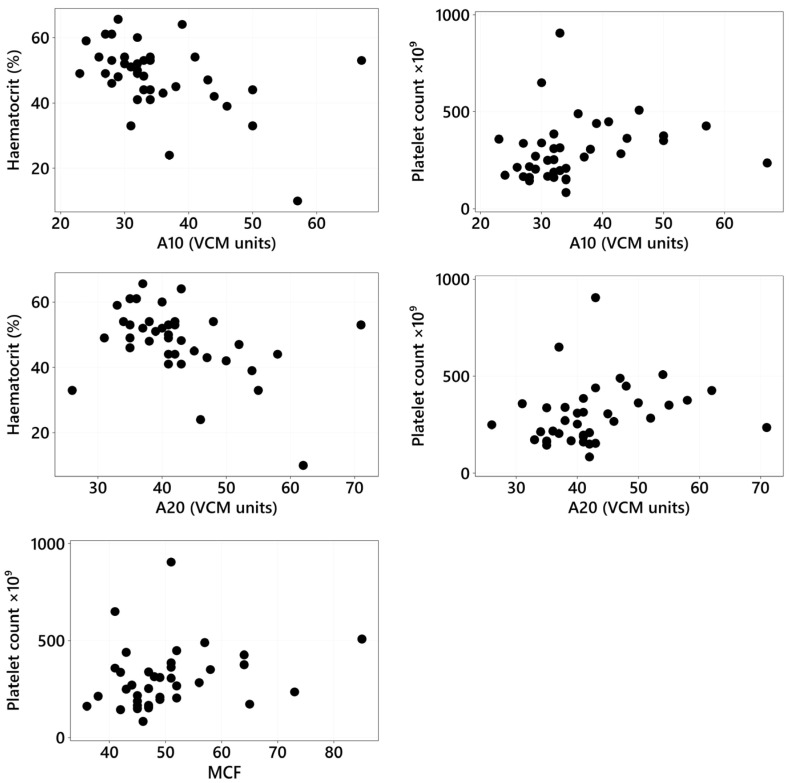
Correlation of hematocrit and platelet count with A10, A20, and MCF where Spearman rank correlation was significant. Abbreviations: A10 = amplitude at 10 min, A20 = amplitude at 20 min, MCF = maximum clot firmness, VCM = viscoelastic coagulation monitor.

The VCM variables (CT, CFT, MCF, α angle, A10, and A20) were correlated with age, sex and neuter status, duration of clinical signs, HCT, platelet count, cobalamin, folate, albumin, and CCECAI scores using Spearman rank correlation for continuous/ordinal data and Fisher exact tests for categorical data. Only the statistically significant correlations are reported in Figure 3. There was a significant weak positive correlation between platelet count and A10 (r_s_ = 0.365, *p* = 0.024), platelet count and A20 (r_s_ = 0.384, *p* = 0.017), and platelet count and MCF (r_s_ = 0.332, *p* = 0.042), and there was a significant moderate negative correlation between HCT and A10 (r_s_ = −0.456, *p* = 0.004) and between HCT and A20 (r_s_ = −0.433, *p* = 0.007) (Figure 3).

Other correlations did not achieve statistical significance, although some were close to reaching significance, including a positive correlation between age and MCF (*p* = 0.068), a negative correlation between albumin and MCF (*p* = 0.078), and a positive correlation between the CCECAI and MCF (*p* = 0.071).

No significant differences were found between VCM variables (CT, CFT, MCF, α angle, A10, and A20) and type of CIE (FRE, IRE, and NRE) based on Kruskal-Wallis tests adjusted for ties.

### 3.5. Suspected Thromboembolic Events

Pulmonary thromboembolism (PTE) was suspected in one patient classified as NRE (case 26, Table 3) that presented with chronic small intestinal diarrhea that was non-responsive to diet, metronidazole, and fenbendazole. The patient was diagnosed with a PLE leading to peripheral edema. This patient underwent bloodwork, fecal and urine analyses, thoracic radiographs, and abdominal ultrasound. Endoscopy was planned for the following day; however, the patient developed acute respiratory distress, suspected secondary to a possible PTE, and subsequently suffered cardiopulmonary arrest. A VCM trace showed that the dog was normocoagulable on presentation.

## 4. Discussion

The primary objective of this study was to describe the coagulation status in dogs diagnosed with CIE using the VCM system, which has become increasingly accessible to veterinary practices. We also aimed to assess whether our findings are in accordance with those from previous studies that utilized TEG, ROTEM, and TEG 6 s. Our results indicate that dogs with CIE tend to exhibit a hypercoagulable state when assessed using the VCM, consistent with prior reports using other coagulation testing methods. Specifically, 81.5% of CIE-affected dogs were hypercoagulable, while 18.5% were normocoagulable. Although our study did not include a group of healthy controls, the use of VCM in healthy canine patients has been previously validated in other research studies [5,13].

Among the different CIE subtypes, hypercoagulability was significantly more frequent in IRE cases, all of which exhibited a hypercoagulable profile, which accounted for 51% of all the hypercoagulable traces. In contrast, normocoagulability was more frequently observed in dogs with FRE, representing 57% of normocoagulable cases. Previously reported prevalences of hypercoagulability in CIE patients when assessed using TEG or ROTEM range between 44.7 and 76% [7,29], and this value increases to 82% when assessed by TEG^®^ 6 s [30] and to 100% in dogs with PLE [6,28]. Similarly, in our cohort, 93% (14/15) of hypoalbuminemic dogs were hypercoagulable. Among the 23 non-PLE patients, 74% (17/23) exhibited hypercoagulability, while 26% were normocoagulable (6/23). No statistically significant difference was observed between albumin levels and coagulation status, consistent with prior reports on dogs with CIE assessed using TEG [7].

Previous studies correlating VCM and ROTEM showed that VCM was not deemed sensitive to detect hypercoagulability, although this could be influenced by the small number of hypercoagulable patients in those studies [13]. In contrast, most of our patients exhibited a hypercoagulable trace, which aligns with the previously reported prevalence of hypercoagulability in dogs with CIE as assessed by TEG [6,7]. One of the main challenges in defining hypercoagulability lies in the absence of a true gold standard test for its diagnosis. In human medicine, the correlation between a hypercoagulable profile on viscoelastic tests and a clinically hypercoagulable state remains under investigation [45]. Therefore, although our findings suggest that VCM may detect coagulation alterations in CIE patients, its clinical utility in evaluating hypercoagulability must be interpreted with caution. Further validation studies, ideally involving comparing VCM results to other viscoelastic methods or clinical outcomes, are necessary before drawing firm conclusions. Viscoelastic coagulation monitor testing offers distinct advantages over TEG and ROTEM, including portability, ease of use, and reliability as a coagulation testing monitor.

It is important to highlight the role of microbial dysbiosis and intestinal inflammation in driving coagulation abnormalities in dogs with CIE. Recent studies have highlighted the interplay between gut microbiota and the immune system, with altered microbiota potentially contributing to systemic inflammation and dysregulated coagulation [46,47]. The exact mechanisms through which gastrointestinal diseases like CIE contribute to abnormal coagulation require more in-depth research, particularly in terms of the effects of cytokines, bacterial endotoxins, and other inflammatory mediators on the coagulation cascade. We only had one dog with MrMRE, and this dog was normocoagulable, so a study with a larger sample size would be required to make conclusions about this form of CIE.

Our second aim was to compare the coagulation status, the VCM variables, and type of CIE with clinical and clinicopathological variables. When comparing clinical and clinicopathological variables with the type of CIE, the CCECAI scores and albumin and cobalamin concentrations were statistically significantly different compared to the type of CIE. Additionally, the correlation of HCT and platelet count with A10, A20, and MCF was statistically significant.

The CCECAI, the disease severity score, was significantly higher in dogs with IRE and NRE when compared with those with FRE, which is in accordance with what has been previously reported [48,49], and although no significant association was found between age and the type of CIE in our population, previous studies have shown that dogs with FRE tend to be younger animals with lower disease severity scores [20,50]. Interestingly, there was no significant association between coagulation status and CCECAI scores, contrasting with recent studies using other hand-held devices [30]. This suggests that the coagulation abnormalities observed in CIE may be more influenced by disease pathophysiology rather than by the severity of clinical signs alone. The lack of a strong association between coagulation status and disease severity highlights the complexity of the pathophysiology of CIE and suggests that coagulation abnormalities may occur independently of the disease severity.

Albumin levels were significantly higher in dogs with FRE compared to those with IRE and NRE. In our cohort, 15 hypoalbuminemic patients were identified, representing 39.5% of all CIE cases included. In the FRE group, only 12.5% of patients had PLE, whereas 60% and 62.5% of NRE and IRE patients had PLE, respectively. The serum albumin concentration can be decreased in dogs with CIE because of gastrointestinal protein loss and is of prognostic value [27,42,48,51,52,53]. Previous studies documented that hypoalbuminemia was more commonly found in NRE and IRE comparatively to FRE patients, likely relating to the severity of the disease [22]. The FRE dogs in our study were less likely to be hypoalbuminemic, but a similar prevalence of hypoalbuminemia was found in both IRE and NRE patients.

We initially hypothesized that patients would present hypercoagulable irrespective of their albumin level, and albumin level was not significantly associated with VCM variables or with overall coagulation status (hypercoagulable vs. normocoagulable). While 93% of hypoalbuminemic dogs were hypercoagulable, 74% of normoalbuminemic dogs were also hypercoagulable. Previous studies have shown that CIE dogs exhibit hypercoagulability, as assessed by TEG, regardless of albumin, like in this study, or antithrombin levels [6,7,29]. One study reported a moderate correlation between serum albumin and MA assessed by TEG [29], but we did not observe any significant correlation between albumin levels and VCM variables.

In human medicine, cobalamin and folate deficiencies and hyperhomocysteneimenia are considered risk factors for hypercoagulability [54,55]. Hyperhomocysteinemia has been identified in cobalamin-deficient dogs [56], which could indicate a potential link to hypercoagulability. In our study, 21 dogs were hypocobalaminemic (6 had FRE, 11 IRE, 3 NRE, and 1 MrMRE), and 11 were hypofolatemic (6 had IRE, 3 FRE, and 2 NRE), which is not unexpected in dogs with CIE, but no significant association was found when comparing these concentrations with the coagulation status or VCM variables. Cobalamin concentrations were significantly higher in dogs with FRE compared to those with NRE or IRE. This aligns with previous studies, which have shown that cobalamin serves as a negative prognostic indicator, generally associated with higher disease severity scores and concurrent hypoalbuminemia in many cases [21,36,42,57,58]. Concurrent hyperfolatemia and hypocobalaminemia were found in one patient (case 28, Table 3) with FRE.

Hypercobalaminemia (>908 ng/L) was detected in six dogs, four of whom were classified as having FRE, one as having IRE, and one as having NRE. This was previously related to severe inflammatory, immune-mediated, and neoplastic conditions [59,60]. In the present study, variable disease severity was found among hypercobalaminemic patients; one had mild disease, three had moderate disease, and two had very severe disease according to the CCECAI scores. These findings suggest that further studies are needed to better understand the significance of hypercobalaminemia in CIE.

Folate levels were decreased in 11 dogs, and 6 of these were concurrently hypocobalaminemic (5 had IRE and 1 had NRE). However, folate did not have a significant association with the type of CIE, coagulation status, or VCM variables, whereas cobalamin showed a significant difference when compared with the type of CIE. This further highlights hypofolatemia as an inferior biomarker in dogs with CIE when compared to hypocobalaminemia, as noted in previous studies [61].

The associations between hematocrit, platelet count, folate, duration of clinical signs, sex and neuter status, and age with the type of CIE were not statistically significant. This lack of significance may be attributed to the relatively small sample size, which could have limited the ability to detect meaningful associations. Hematocrit and platelet count can influence both the rate of clot formation and clot strength [2,9,44,53,56,62]. There was a significant positive correlation between the platelet count and MCF, A10, and A20, which are three different measurements of clot firmness, indicating that higher platelet counts are associated with increased clot firmness. This finding is not surprising given the importance of platelets in clot formation, and MCF represents a phase of coagulation that occurs at the platelet surface and is therefore influenced by platelet concentration [63,64].

Anemia has been linked to hypercoagulable states [9,29,65]. An important consideration in interpreting viscoelastometric results is the potential impact of anemia on clot dynamics. Anemia has been shown to affect viscoelastic parameters, often resulting in a hypercoagulable profile. This effect is likely due to the increased proportion of plasma in whole blood samples from anemic patients, which can lead to enhanced clot formation independent of a true hypercoagulable state [66]. Therefore, the presence of anemia in some of our CIE patients may act as a confounding factor, and this must be considered when interpreting VCM results. In our study, a significant moderate negative correlation was found between HCT, A10, and A20, which measure clot amplitude at 10 and 20 min, respectively, and reflect clot firmness, indicating superior clot firmness in patients with a lower HCT. These results suggest that lower hematocrit levels are associated with superior clot firmness, reinforcing the connection between anemia and hypercoagulability. In the hypercoagulable group, two anemic patients were included (cases 14 and 22, Table 3), both hypercoagulable. One dog (case 22, Table 3) had a moderate non-regenerative anemia, suspected to be secondary to chronic disease, and one had a severe regenerative anemia (case 14, Table 3) secondary to melena and hematochezia, which required a packed red blood cell transfusion after VCM testing. Anemia could have potentially influenced the results, leading to a hypercoagulable state through compensatory mechanisms, such as increased platelet count and fibrinogen levels, which contribute to enhanced clot formation. Additionally, anemia of chronic disease may also lead to a hypercoagulable state due to elevated levels of procoagulant factors, such as fibrinogen, and alterations in the balance of the coagulation system. These changes can, in some cases, increase the risk of thrombosis [9,29,65].

The most frequent abnormalities observed were increased A20 in 37 dogs, elevated MCF in 29 dogs, and increased A10 in 26 dogs. Specifically, A10 and A20 reflect clot firmness 10 and 20 min after clot initiation, respectively, and are influenced by fibrin polymerization and platelet–fibrin interactions. Their consistent elevation suggests accelerated clot formation and increased clot strength at early and intermediate stages of coagulation. Similarly, MCF, which represents the final overall strength of the clot, was also elevated in most dogs, indicating enhanced clot stability—often due to elevated fibrinogen levels, increased platelet function, or both [2]. Together, the predominant elevation of A10, A20, and MCF—parameters closely linked to clot strength and firmness—strongly supports the presence of a hypercoagulable profile in the majority of dogs with CIE.

Our third aim was to evaluate the presence of thromboembolic events and whether these could be anticipated by VCM results. One of the dogs in our study showed clinical signs compatible with possible pulmonary thromboembolism (PTE) during the observation period. This finding underscores the need for ongoing monitoring of coagulation status in dogs with CIE, especially those with PLE or other predisposing factors for thromboembolism. The patient suspected to have possibly developed pulmonary thromboembolism (case 26, Table 3) was diagnosed with very severe PLE according to the CCECAI score, leading to peripheral pitting edema, and was found to be normocoagulable on presentation. Upon admission, point-of-care abdominal and thoracic ultrasound were conducted, showing no abdominal, pleural, or pericardial effusions. An abdominal ultrasound showed moderate, diffuse thickening of the duodenal mucosa and colon, but no loss of layering, masses, or foreign bodies were visualized. Thoracic radiographs found traces of pleural effusion and a caudodorsal interstitial-to-alveolar pattern. The patient developed tachypnea, dyspnea, hemoptysis, and hemorrhagic small intestinal diarrhea, resulting in cardiopulmonary arrest. This led to the suspicion of possible pulmonary thrombosis and disseminated intravascular coagulation (DIC), which can present in different forms. Typically, DIC is initiated by a prothrombotic or hypercoagulable state, which may transiently progress through a balanced phase where normocoagulability is observed, and eventually evolves into a decompensated state characterized by hypocoagulability due to the consumption of coagulation factors and platelets, often accompanied by hypofibrinolysis [67,68,69]. Alternatively, a hyperfibrinolytic form of DIC may occur, marked by excessive fibrinolysis and severe hemorrhage, without preceding coagulation factor consumption [70]. The normocoagulable trace observed in this patient may reflect a transient stage in the progression of an underlying coagulopathy, such as evolving DIC, rather than indicating the absence of coagulation abnormalities. Although the patient exhibited clinical signs suggestive of PTE, this was more likely a consequence of a preceding hypercoagulable state rather than a cause of the normocoagulable profile. This patient did not undergo endoscopy and gastrointestinal biopsies to confirm a diagnosis of CIE leading to PLE, but non-responsive enteropathy was suspected based on clinical presentation and response to treatment.

Hypercoagulability represents a risk from thromboembolic events, but the exact incidence is yet unknown and non-predictable [71]. Thromboembolism has been demonstrated in human and canine patients suffering from CIE; PLE is associated with a moderate to strong risk of arterial or venous thrombosis in 2.6% of cases, more commonly pulmonary thromboembolism, as suspected in our case, which suggests that this is a relevant complication of PLE [65,72,73,74,75,76,77,78]. Many of the patients in previous studies documenting thrombosis were also receiving corticosteroids, so multiple factors could have affected the coagulation status. Additionally, many studies were not designed to assess thromboembolic events, so the prevalence could be underestimated. Given the hypercoagulability in CIE dogs, particularly in the IRE group, it is likely that these dogs are at an increased risk of developing thromboembolic events, particularly those with PLE. Future studies with larger sample sizes and longer follow-up periods will be essential to determine whether abnormal coagulation profiles, as measured by the VCM, can predict thromboembolic events in dogs with CIE. Additionally, clinical trials assessing the efficacy of anticoagulant therapies in managing coagulation abnormalities and preventing thromboembolic complications in these dogs could provide valuable insights for improving patient outcomes. Antithrombotic therapy has been recommended for dogs with PLE, unless risks associated with therapy outweigh individual benefits [28,34,76]. Treatment is normally reserved for patients with PLE and/or documented emboli [34,79], but the use of prophylactic antithrombotic therapy in CIE patients warrants further investigations. In our study, 7 of the 15 patients with PLE were prescribed antithrombotic prophylactic therapy with clopidogrel (loading dose of 4–10 mg/kg once daily on the first day of therapy, followed by 2 mg/kg once daily as a maintenance dose), they were all classified as having IRE-PLE, and all of them were moderately to severely hypoalbuminemic.

The reported prevalence of FRE among dogs with CIE is reported to be around 50–65% [48,50]. Our cohort included 16 FRE patients, 42% of all included patients, which is lower than the previously reported prevalence. Of these, 15 responded to the first diet trial, 12 of them responded to a hydrolyzed protein diet, and 3 of them responded to a low-fat diet (2 on a gastrointestinal low-fat diet and one on a home-cooked low-fat diet), while 1 patient required two diet trials (first a hydrolyzed diet and then a novel protein diet to resolve the clinical signs), reiterating the importance of more than one diet trial if required [20].

From the IRE group, all the patients were prescribed prednisolone therapy (dose range of 1–2 mg/kg orally once daily), with three of them requiring a second immunosuppressive medication, such as cyclosporin (*n* = two dogs, one with IRE-PLE and one with IRE, with a dose range of 5–10 mg/kg orally once daily) or chlorambucil (*n* = one dog with IRE-PLE, with a dose of 2 mg/m^2^ once daily). Four dogs were classified as having IRE and did not undergo endoscopy and biopsies due to financial concerns but responded well to immunosuppressive therapy.

The limitations of this study include its retrospective nature limiting the uniformity and design of the study. Regarding VCM testing, the device is sensitive to external factors such as movement and vibrations [80]. Therefore, variations in how the device is handled, especially when operated by different laboratory technicians, could impact the consistency of the results. Secondly, clinician-dependent decisions affected the standardization of therapy and could have influenced the classification of cases. Since recent updated guidelines on classification in dogs with CIE included the category of MrMRE [20], and a large proportion of our cases presented before this, a low number of MrMRE patients were encountered. Additionally, while we used the VCM as a point-of-care device, further validation studies comparing VCM results with other coagulation tests (e.g., TEG, ROTEM, TEG 6 s, and standard coagulation tests) in larger, diverse populations of dogs with CIE are needed. We used the equipment reference intervals for VCM analysis, but using a control group of healthy dogs will provide additional value in future studies. One last limitation is that four dogs in the IRE group did not undergo endoscopy and biopsies due to financial concerns, but a good response was seen to immunosuppressive therapy during the available follow-up period. Additionally, two dogs that were classified as having NRE died within 24–36 h of hospitalization and also did not undergo endoscopy and biopsies.

## 5. Conclusions

In conclusion, this study found that hypercoagulability is common in dogs with CIE, particularly in those with IRE and NRE, aligning with previous research. Additionally, coagulation abnormalities were not significantly associated with disease severity scores, suggesting that they are more influenced by the underlying pathophysiology rather than clinical signs alone. Cobalamin levels were significantly higher in FRE patients, while hypoalbuminemia was more common in IRE and NRE patients. Hypercobalaminemia was detected in several patients, particularly those with FRE, although no statistical difference was found, and further studies are required to understand its clinical significance in CIE. Although this study identified a proportion of CIE dogs with a hypercoagulable viscoelastic profile, the limited number of observed or suspected thromboembolic events precludes any conclusion about a direct correlation between hypercoagulability and thrombotic risk. It is important to recognize that thromboembolism is a multifactorial process, influenced not only by coagulation status but also by factors such as endothelial injury and blood flow alterations [67]. Further research with larger case numbers and confirmed thromboembolic events is needed to clarify the clinical relevance of viscoelastic hypercoagulability in this context.

Our study suggests that VCM could be a reliable coagulation testing monitor to be used in the clinical setting in patients with CIE, with similar findings to other published studies using different methodologies in this disease [6,7,30]. The VCM has the advantages of being a portable device and being easy to use, allowing for rapid, real-time hemostasis assessment, including clot formation, retraction, and lysis, from a small whole-blood sample. Thromboelastography counts on evidence-based guidelines for its performance in companion animals [41,81]; however, such guidelines are lacking for VCM analysis, and further research is warranted to further validate this method.

## Figures and Tables

**Table 1:** Summary of clinical signs.

Clinical Sign	Number of Cases (*n*)	Percentage of Total (%)
Vomiting	28	73.68
Diarrhea	27	71.05
Reduced appetite	18	47.37
Weight loss	11	28.95
Hematochezia	11	28.95
Lethargy	8	21.05
Borborygmi	7	18.42
Abdominal discomfort	6	15.79
Polyuria/polydipsia	4	10.53
Regurgitation	3	7.89
Melena	2	5.26
Ascites	2	5.26
Hypersalivation	1	2.63
Peripheral edema	1	2.63
Flatulence	1	2.63

**Table 2:** Summary of VCM variables and VCM results for all included dogs.

Case	CT (RI 241–470 s)	CFT (104–266 s)	α Angle (RI 43–64 Degrees)	A10 (RI 16–30 VCM Units)	A20 (RI 22–28 VCM Units)	MCF (RI 29–44)	VCM Result
1	393	124	60	34 *	43 *	47 *	Hypercoagulable
2	319	128	64	28	35 *	36	Normocoagulable
3	378	163	56	27	35 *	45 *	Hypercoagulable
4	426	127	61	31*	39 *	47 *	Hypercoagulable
5	345	172	55	24	33 *	65 *	Hypercoagulable
6	360	156	58	29	38 *	44	Normocoagulable
7	392	108	65 *	32 *	41 *	45 *	Hypercoagulable
8	382	80 *	67 *	44 *	50 *	51 *	Hypercoagulable
9	314	97 *	64	43 *	52 *	56 *	Hypercoagulable
10	306	110	65 *	32 *	40 *	47 *	Hypercoagulable
11	206 *	108	63	30	38 *	47 *	Hypercoagulable
12	305	104	63	38 *	45 *	51 *	Hypercoagulable
13	333	126	50	31 *	26	43	Normocoagulable
14	179 *	56 *	72 *	57 *	62 *	64 *	Hypercoagulable
15	106 *	111	59	50 *	58 *	64 *	Hypercoagulable
16	283	131	58	32 *	41 *	51 *	Hypercoagulable
17	320	160	51	36 *	47 *	57 *	Hypercoagulable
18	303	125	60	33 *	41 *	48 *	Hypercoagulable
19	281	94 *	69 *	34 *	42 *	46 *	Hypercoagulable
20	284	122	59	33 *	41 *	49 *	Hypercoagulable
21	262	66 *	69 *	67 *	71 *	73 *	Hypercoagulable
22	425	125	59	37 *	46 *	52 *	Hypercoagulable
23	362	152	55	32 *	41 *	47 *	Hypercoagulable
24	333	147	54	30	37 *	41	Normocoagulable
25	326	80 *	73 *	34 *	42 *	45 *	Hypercoagulable
26	464	196	47	26	34 *	38	Normocoagulable
27	354	121	61	33 *	43 *	51 *	Hypercoagulable
28	169 *	117	62	28	35 *	42	Hypercoagulable
29	310	120	65 *	29	37 *	52 *	Hypercoagulable
30	350	86 *	65 *	50 *	55 *	58 *	Hypercoagulable
31	186 *	54 *	76 *	46 *	54 *	85 *	Hypercoagulable
32	233 *	80 *	70 *	41 *	48 *	52 *	Hypercoagulable
33	287	106	66 *	34 *	42 *	49 *	Hypercoagulable
34	417	165	54	27	35 *	42	Normocoagulable
35	330	146	58	28	36 *	45 *	Hypercoagulable
36	267	110	66 *	32 *	40 *	49 *	Hypercoagulable
37	414	175	56	23	31 *	41	Normocoagulable
38	255	75 *	73 *	39 *	43 *	43	Hypercoagulable

* Asterisks denote values outside of reference interval. Abbreviations: A10 = amplitude at 10 min, A20 = amplitude at 20 min, CT = clot time, CFT = clot formation time, MCF = maximum clot firmness, VCM = viscoelastic coagulation monitor.

**Table 3:** Summary of clinical and clinicopathological results for all included patients.

Case	Age (months)	Sex and Neuter Status	Duration of Clinical Signs (weeks)	Hct (37–55%)	Platelet Count (140–700 × 10^9^/L)	Albumin (24–40 g/L)	Cobalamin (400–907 ng/L)	Folate (7.7–24.4 ng/L)	CCECAI Score	VCM Result	CIE Type
1	68	MN	4	41	154	28	171 *	18	5–mild	Hypercoagulable	IRE
2	32	FN	40	53	162	33	448	10	6—moderate	Normocoagulable	FRE
3	99	FN	16	61	166	26	185 *	7 *	8—moderate	Hypercoagulable	IRE
4	17	ME	5	51	167	31	1000 *	11	6—moderate	Hypercoagulable	FRE
5	107	FN	16	59	173	32	593	8	7—moderate	Hypercoagulable	FRE
6	135	FN	20	48	271	38	265 *	21	8—moderate	Normocoagulable	FRE
7	112	FN	8	41	189	32	413	6 *	8—moderate	Hypercoagulable	IRE
8	18	FN	16	42	363	16 *	1000 *	12	12—very severe	Hypercoagulable	FRE-PLE
9	108	FN	4	47	284	11 *	217 *	5 *	14—very severe	Hypercoagulable	IRE-PLE
10	15	FN	4	52	253	23 *	140 *	6 *	6—moderate	Hypercoagulable	IRE-PLE
11	79	ME	15	54	339	33	500	5 *	6—moderate	Hypercoagulable	FRE
12	76	MN	60	45	307	33	1216 *	13	8—moderate	Hypercoagulable	FRE
13	6	FE	12	37	250	33	213 *	14	5—mild	Normocoagulable	MrMRE
14	84	ME	5	10 *	427	32	149 *	10	9—severe	Hypercoagulable	NRE
15	150	FE	8	44	376	34	493	16	6—moderate	Hypercoagulable	IRE
16	126	FN	9	50	386	16 *	164 *	23	6—moderate	Hypercoagulable	IRE-PLE
17	111	FN	16	43	490	18 *	385 *	18	9—severe	Hypercoagulable	IRE-PLE
18	85	MN	9	44	314	12 *	140 *	18	10—severe	Hypercoagulable	IRE-PLE
19	103	MN	14	54	84 *	37	1223 *	16	8—moderate	Hypercoagulable	IRE
20	36	ME	60	53	197	30	923 *	12	5—mild	Hypercoagulable	FRE
21	141	FN	14	53	236	38	287 *	8	3—insignificant	Hypercoagulable	FRE
22	53	MN	8	24 *	267	22 *	273 *	23	4—mild	Hypercoagulable	FRE-PLE
23	90	FN	5	49	161	33	141 *	8	6—moderate	Hypercoagulable	FRE
24	47	FN	10	52	650	26	218 *	6 *	10—severe	Normocoagulable	NRE
25	20	FN	3	44	150	17 *	167 *	10	13—very severe	Hypercoagulable	IRE-PLE
26	118	MN	3	54	214	12 *	97 *	4 *	12—very severe	Normocoagulable	NRE-PLE
27	103	ME	3	48	906 *	13 *	N/A	N/A	16—very severe	Hypercoagulable	IRE-PLE
28	27	ME	4	46	144	32	315 *	30.6 *	6—moderate	Hypercoagulable	FRE
29	138	FN	6	65	205	19 *	228 *	9.4	8—moderate	Hypercoagulable	IRE-PLE
30	74	ME	4	37	351	9 *	182 *	4.2 *	14—very severe	Hypercoagulable	IRE-PLE
31	96	MN	24	39	509	15 *	1000 *	8	13—very severe	Hypercoagulable	NRE-PLE
32	112	FN	3	54	449	12 *	224 *	7.4 *	10—severe	Hypercoagulable	IRE-PLE
33	156	MN	4	53	209	37	485	9.8	4—mild	Hypercoagulable	IRE
34	136	ME	6	49	337	32	565	7.3 *	5—mild	Normocoagulable	FRE
35	84	FN	3	61	217	34	246 *	9.6	7—moderate	Hypercoagulable	FRE
36	16	FE	3	60	310	16 *	745	10.3	10—severe	Hypercoagulable	NRE-PLE
37	46	ME	8	49	359	34	797	15.3	5—mild	Normocoagulable	FRE
38	91	FN	16	64	440	34	700	2.2 *	3—insignificant	Hypercoagulable	FRE

* Asterisks denote values outside of reference interval. Abbreviations: CCECAI = canine chronic enteropathy clinical activity index, CIE = chronic inflammatory enteropathy, FE = female entire, FN = female neutered, FRE = food-responsive enteropathy, HCT = hematocrit, IRE = immunosuppressive-responsive enteropathy, ME = male entire, MN = male neutered, MrMRE = microbiota-related modulation-responsive enteropathy, NRE = non-responsive enteropathy, PLE = protein-losing enteropathy, VCM = viscoelastic coagulation monitor.

**Table 4:** Minimum and maximum values of clinical and clinicopathological results by type of CIE.

CIE Type	CIE Subtype	Number of Dogs	Age (months)	Duration of Signs (weeks)	Hct (%)	Platelet Count (×10^9^/L)	Albumin (g/L)	Cobalamin (ng/L)	Folate (ng/L)	CCECAI Score
FRE	PLE	2	18–53	8–16	24–42	267–363	16–22	273–1000	12–23	4–12
	Non-PLE	14	17–141	3–60	45–64	144–440	31–38	141–1216	2.2–30.6	3–8
IRE	PLE	10	15–138	4–16	43–65	150–906	9–23	140–385	4.2–26	6–16
	Non-PLE	6	68–156	4–16	41–61	84–376	26–37	171–1223	6–18	4–8
MrMRE	Non-PLE	1	68	12	41	154	28	213	18	5
NRE	PLE	3	47–84	3–24	39–60	427–650	12–16	97–1000	4–10.3	10–13
	Non-PLE	2	16–118	5–10	10–52	214–509	26–32	149–218	6–10	6–10

Abbreviations: CCECAI = canine chronic enteropathy clinical activity index, CIE = chronic inflammatory enteropathy, FRE = food-responsive enteropathy, HCT = hematocrit, IRE = immunosuppressive-responsive enteropathy, MrMRE = microbiota-related modulation-responsive enteropathy, NRE = non-responsive enteropathy, PLE = protein-losing enteropathy.

**Table 5:** Summary of coagulation status and type of CIE.

CIE Subtype	Number of Dogs (%)	Coagulation Status
FRE	16 (42.1%)	12 hypercoagulable (75%) 4 normocoagulable (25%)
IRE	16 (42.1%)	16 hypercoagulable (100%) 0 normocoagulable (0%)
MrMRE	1 (2.6%)	1 normocoagulable (100%)
NRE	5 (13.2%)	3 hypercoagulable (60%) 2 normocoagulable (40%)

Abbreviations: CIE = chronic inflammatory enteropathy, FRE = food-responsive enteropathy, IRE = immunosuppressive-responsive enteropathy, MrMRE = microbiota-related modulation-responsive enteropathy, NRE = non-responsive enteropathy.

**Table 6:** Median (minimum–maximum) values of albumin, cobalamin, and CCECAI score by type of CIE, analyzed using Kruskal-Wallis tests adjusted for ties.

Parameter	FRE	IRE	NRE	*p*-Value
Albumin (g/L)	33 (16–38)	18 (9–37)	16 (12–32)	0.005
Cobalamin (ng/L)	532 (141–1216)	217 (140–1223)	218 (97–1000)	0.028
CCECAI	6 (3–12) (insignificant to very severe)	8 (4–16) (mild to very severe)	10 (7–13) (severe to very severe)	0.004

Abbreviations: CCECAI = canine chronic enteropathy clinical activity index, FRE = food-responsive enteropathy, IRE = immunosuppressive-responsive enteropathy, NRE = non-responsive enteropathy, PLE = protein-losing enteropathy.

## Data Availability

All data analyzed during this study are included in this published article.

## References

[1] Entegrion I. VCM Vet—Real Time Hemostasis Assessment. https://vcmvet.com/.

[2] Burton A.G., Jandrey K.E. (2020). Use of Thromboelastography in Clinical Practice. Vet. Clin. N. Am.-Small Anim. Pract..

[3] Rosati T., Jandrey K.E., Burges J.W., Kent M.S. (2020). Establishment of a Reference Interval for a Novel Viscoelastic Coagulometer and Comparison with Thromboelastography in Healthy Cats. Vet. Clin. Pathol..

[4] Buriko Y., Chalifoux N.V., Clarkin-Breslin R., Silverstein D.C. (2023). Comparison of a Viscoelastic Point-of-Care Coagulation Monitor with Thromboelastography in Sick Dogs with Hemostatic Abnormalities. Vet. Clin. Pathol..

[5] Buriko Y., Drobatz K., Silverstein D.C. (2020). Establishment of Normal Reference Intervals in Dogs Using a Viscoelastic Point-of-Care Coagulation Monitor and Its Comparison with Thromboelastography. Vet. Clin. Pathol..

[6] Goodwin L.V., Goggs R., Chan D.L., Allenspach K. (2011). Hypercoagulability in Dogs with Protein-Losing Enteropathy. J. Vet. Intern. Med..

[7] Dixon A., Hall E.J., Adamantos S., Kathrani A., McGrath C., Black V. (2021). Hypercoagulability in Dogs with Chronic Enteropathy and Association with Serum Albumin Concentration. J. Vet. Intern. Med..

[8] Min S., Wesselowski S.R., Nabity M.B., Yankin I. (2024). Pulmonary Hypertension Is Associated with Hypocoagulability in Dogs: A Retrospective Analysis of 66 Cases (2013–2021). Am. J. Vet. Res..

[9] Dionne T.L., Ishak A.M., Cochran L.A. (2023). Point-of-Care Global Coagulation Assay Parameters in Normal Dogs and Dogs with Primary Immune-Mediated Hemolytic Anemia. J. Vet. Emerg. Crit. Care.

[10] Wang W.H., Lynch A.M., Balko J.A., Duffy D.J., Robertson J.B., Posner L.P. (2022). Point-of-Care Viscoelastic Coagulation Assessment in Healthy Dogs during the Perianesthetic Period. BMC Vet. Res..

[11] Morris L., Galezowski A., Atilla A., Menard J. (2024). Short-duration peripherally inserted central catheters do not alter viscoelastic parameters in healthy dogs. Canine Vet. J..

[12] Chang J., Jandrey K.E., Burges J.W., Kent M.S. (2021). Comparison of Healthy Blood Donor Greyhounds and Non-Greyhounds Using a Novel Point-of-Care Viscoelastic Coagulometer. J. Vet. Emerg. Crit. Care.

[13] Hennink I., Peters L., van Geest G., Adamik K.N. (2023). Evaluation of a Viscoelastic Coagulation Monitoring System (VCM Vet^®^) and Its Correlation with Thromboelastometry (ROTEM^®^) in Diseased and Healthy Dogs. Animals.

[14] Moses I.A., Hallowell T.C., Johnson J.A. (2024). Feline Cardiomyopathy Patients Do Not Exhibit Evidence of Hypercoagulability Using a Novel Viscoelastic Coagulation Monitor. Am. J. Vet. Res..

[15] Moreno D., Cosford K., Snead E., Carr A. (2024). Assessment of Hemostasis in Hyperthyroid and Euthyroid Cats Using Two Viscoelastic Assays and Platelet Aggregometry. J. Vet. Intern. Med..

[16] Fudge J.M., Cano K.S., Page B., Jeffery U. (2021). Comparison of Viscoelastic Test Results from Blood Collected near Simultaneously from the Jugular and Saphenous Veins in Cats. J. Feline Med. Surg..

[17] Yozova I.D., Kent M.S., Jandrey K.E. (2023). Effects of a Single Subcutaneous Dose of Enoxaparin on Veterinary Viscoelastic Coagulation Monitor Variables in Healthy Cats: Double Blind, Placebo Controlled Cross-over Trial. J. Vet. Intern. Med..

[18] Fudge J.M., Page B., Mackrell A., Lee I., Jeffery U. (2021). Blood Loss and Coagulation Profile in Pregnant and Non-Pregnant Queens Undergoing Elective Ovariohysterectomy. J. Feline Med. Surg..

[19] Dandrieux J.R.S. (2016). Inflammatory Bowel Disease versus Chronic Enteropathy in Dogs: Are They One and the Same?. J. Small Anim. Pract..

[20] Dupouy-Manescau N., Méric T., Sénécat O., Drut A., Valentin S., Leal R.O., Hernandez J. (2024). Updating the Classification of Chronic Inflammatory Enteropathies in Dogs. Animals.

[21] Dandrieux J.R.S., Mansfield C.S. (2019). Chronic Enteropathy In Canines: Prevalence, Impact And Management Strategies. Vet. Med. Res. Rep..

[22] Holmberg J., Pelander L., Ljungvall I., Harlos C., Spillmann T., Häggström J. (2022). Chronic Enteropathy in Dogs-Epidemiologic Aspects and Clinical Characteristics of Dogs Presenting at Two Swedish Animal Hospitals. Animals.

[23] Simpson K.W., Jergens A.E. (2011). Pitfalls and Progress in the Diagnosis and Management of Canine Inflammatory Bowel Disease. Vet. Clin. N. Am.-Small Anim. Pract..

[24] Berghoff N., Steiner J.M. (2011). Laboratory Tests for the Diagnosis and Management of Chronic Canine and Feline Enteropathies. Vet. Clin. N. Am.-Small Anim. Pract..

[25] Salavati Schmitz S., Gow A., Bommer N., Morrison L., Mellanby R. (2019). Diagnostic Features, Treatment, and Outcome of Dogs with Inflammatory Protein-Losing Enteropathy. J. Vet. Intern. Med..

[26] Peterson P.B., Willard M.D. (2003). Protein-Losing Enteropathies. Vet. Clin. N. Am. Small Anim. Pract..

[27] Dossin O., Lavoué R. (2011). Protein-Losing Enteropathies in Dogs. Vet. Clin. N. Am. Small Anim. Pract..

[28] Nagahara T., Ohno K., Nagao I., Nakagawa T., Yokoyama N., Ohmi A., Goto-Koshino Y., Chambers J.K., Uchida K., Tomiyasu H. (2021). Changes in the Coagulation Parameters in Dogs with Protein-Losing Enteropathy between before and after Treatment. J. Vet. Med. Sci..

[29] Wennogle S.A., Olver C.S., Shropshire S.B. (2021). Coagulation Status, Fibrinolysis, and Platelet Dynamics in Dogs with Chronic Inflammatory Enteropathy. J. Vet. Intern. Med..

[30] Barth S.I., DeMonaco S.M., Conner B.J., Wilkinson A.R. (2025). Hypercoagulability Identified in Dogs with Chronic Enteropathy Using a Point-of-care Viscoelastic Assay. J. Small Anim. Pract..

[31] Rose L.J., Dunn M.E., Allegret V., Bédard C. (2011). Effect of Prednisone Administration on Coagulation Variables in Healthy Beagle Dogs. Vet. Clin. Pathol..

[32] Brainard B.M., Meredith C.P., Callan M.B., Budsber S.C., Shofer F.S., Driessen B., Otto C.M. (2007). Changes in Platelet Function, Hemostasis, and Prostaglandin Expression after Treatment with Non Steroidal Anti-Inflammatory Drugs with Various COX Selectivities in Dogs. Am. J. Vet. Res..

[33] Donahue S.M., Otto C.M. (2005). Thromboelastography: A Tool for Measuring Hypercoagulability, Hypocoagulability, and Fibrinolysis. J. Vet. Emerg. Crit. Care.

[34] De Laforcade A., Bacek L., Blais M.C., Boyd C., Brainard B.M., Chan D.L., Cortellini S., Goggs R., Hoareau G.L., Koenigshof A. (2022). 2022 Update of the Consensus on the Rational Use of Antithrombotics and Thrombolytics in Veterinary Critical Care (CURATIVE) Domain 1-Defining Populations at Risk. J. Vet. Emerg. Crit. Care.

[35] Gold A.J., Langlois D.K., Refsal K.R. (2016). Evaluation of Basal Serum or Plasma Cortisol Concentrations for the Diagnosis of Hypoadrenocorticism in Dogs. J. Vet. Intern. Med..

[36] Kather S., Grützner N., Kook P.H., Dengler F., Heilmann R.M. (2020). Review of Cobalamin Status and Disorders of Cobalamin Metabolism in Dogs. J. Vet. Intern. Med..

[37] Cobalamin Information-Gastrointestinal Laboratory. https://vetmed.tamu.edu/gilab/research/cobalamin-information/.

[38] Serum Cobalamin (Vitamin B12) and Folate-Gastrointestinal Laboratory. https://vetmed.tamu.edu/gilab/service/assays/b12folate/.

[39] Lavanya M., Jayanthi C., Alexandria M., Janani V. (2019). Platelet Estimation by Manual and Automated Methods. Ann. Pathol. Lab. Med..

[40] Baird T.N., Zersen K.M., Guillaumin J. (2024). Point-of-Care Viscoelastic Coagulation Monitoring Device Shows Promise for Informing Resuscitation Strategies in a Canine Hemorrhagic Shock Model. Am. J. Vet. Res..

[41] Hanel R.M., Chan D.L., Conner B., Gauthier V., Holowaychuk M., Istvan S., Walker J.M., Wood D., Goggs R., Wiinberg B. (2014). Systematic Evaluation of Evidence on Veterinary Viscoelastic Testing Part 4: Definitions and Data Reporting. J. Vet. Emerg. Crit. Care.

[42] Allenspach K., Wieland B., Gröne A., Gaschen F. (2007). Chronic Enteropathies in Dogs: Evaluation of Risk Factors for Negative Outcome. J. Vet. Intern. Med..

[43] Jergens A.E., Schreiner C.A., Frank D.E., Niyo Y., Ahrens F.E., Eckersall P.D., Benson T.J., Evans R. (2003). A Scoring Index for Disease Activity in Canine Inflammatory Bowel Disease. J. Vet. Intern. Med..

[44] DeBerry J., Côté E., Ettinger S.J., Feldman E.C. (2024). Hypoproteinemia and Hyperproteinemia. Ettinger’s Textbook of Veterinary Internal Medicine.

[45] Harahsheh Y., Ho K.M. (2018). Use of Viscoelastic Tests to Predict Clinical Thromboembolic Events: A Systematic Review and Meta-Analysis. Eur. J. Haematol..

[46] Eissa N., Kittana H., Gomes-Neto J.C., Hussein H. (2019). Mucosal Immunity and Gut Microbiota in Dogs with Chronic Enteropathy. Res. Vet. Sci..

[47] Maiuolo J., Carresi C., Gliozzi M., Mollace R., Scarano F., Scicchitano M., Macrì R., Nucera S., Bosco F., Oppedisano F. (2022). The Contribution of Gut Microbiota and Endothelial Dysfunction in the Development of Arterial Hypertension in Animal Models and in Humans. Int. J. Mol. Sci..

[48] Allenspach K., Culverwell C., Chan D. (2016). Long-Term Outcome in Dogs with Chronic Enteropathies: 203 Cases. Vet. Record..

[49] Münster M., Hörauf A., Bilzer T. (2006). Assessment of Disease Severity and Outcome of Dietary, Antibiotic, and Immunosuppressive Interventions by Use of the Canine IBD Activity Index in 21 Dogs with Chronic Inflammatory Bowel Disease. Berl. Munch. Tierarztl. Wochenschr..

[50] Jackson M.I., Gaschen F.P., Salavati S., Jergens A.E. (2022). Canine Chronic Enteropathy—Current State-of-the-Art and Emerging Concepts. Front. Vet. Sci..

[51] Heilmann R.M., Steiner J.M., Romy Heilmann C.M. (2018). Clinical Utility of Currently Available Biomarkers in Inflammatory Enteropathies of Dogs. J. Vet. Intern. Med..

[52] Heilmann R.M., Otoni C.C., Jergens A.E., Grützner N., Suchodolski J.S., Steiner J.M. (2014). Systemic Levels of the Anti-Inflammatory Decoy Receptor Soluble RAGE (Receptor for Advanced Glycation End Products) Are Decreased in Dogs with Inflammatory Bowel Disease. Vet. Immunol. Immunopathol..

[53] Wennogle S.A., Priestnall S.L., Webb C.B. (2017). Histopathologic Characteristics of Intestinal Biopsy Samples from Dogs With Chronic Inflammatory Enteropathy With and Without Hypoalbuminemia. J. Vet. Intern. Med..

[54] Owczarek D., Cibor D., Głowacki M.K., Rodacki T., Mach T. (2014). Inflammatory Bowel Disease: Epidemiology, Pathology and Risk Factors for Hypercoagulability. World J. Gastroenterol..

[55] Danese S., Papa A., Saibeni S., Repici A., Malesci A., Vecchi M. (2007). Inflammation and Coagulation in Inflamma-tory Bowel Disease: The Clot Thickens. Am. J. Gastroenterol..

[56] Rossi G., Breda S., Giordano A., Pengo G., Dall’Ara P., Rossi G., Bo S., Paltrinieri S. (2013). Association between Hypocobalaminaemia and Hyperhomocysteinaemia in Dogs. Vet. Rec..

[57] Toresson L., Suchodolski J.S., Spillmann T., Lopes B.C., Shih J., Steiner J.M., Pilla R. (2023). The Intestinal Microbiome in Dogs with Chronic Enteropathies and Cobalamin Deficiency or Normocobalaminemia-A Comparative Study. Animals.

[58] Volkmann M., Steiner J.M., Fosgate G.T., Zentek J., Hartmann S., Kohn B. (2017). Chronic Diarrhea in Dogs–Retrospective Study in 136 Cases. J. Vet. Intern. Med..

[59] Henry P.M.N., Williams T.L. (2023). Prevalence of Neoplasia and Concurrent Diseases in Dogs and Cats with Hypercobalaminemia: A Retrospective Case–Control Study. Vet. Clin. Pathol..

[60] Da Riz F., Higgs P., Ruiz G. (2021). Diseases Associated with Hypercobalaminemia in Dogs in United Kingdom: A Retrospective Study of 47 Dogs. Can. Vet. J..

[61] Ullal T.V., Marks S.L., Huebner S.N., Taylor S.L., Shelley C.D. (2023). Association of Folate Concentrations with Clinical Signs and Laboratory Markers of Chronic Enteropathy in Dogs. J. Vet. Intern. Med..

[62] Lehmann E.L. (2006). Nonparametrics: Statistical Methods Based on Ranks.

[63] Smith S.A., McMichael M.A., Gilor S., Galligan A.J., Hoh C.M. (2012). Correlation of Hematocrit, Platelet Concentration and Plasma Coagulation Factors with Results of TEM in Canine Whole Blood Samples. Am. J. Vet. Res..

[64] Smith S.A. (2009). The Cell-Based Model of Coagulation: State-Of-The-Art Review. J. Vet. Emerg. Crit. Care.

[65] Fenty R.K., De Laforcade A.M., Shaw S.P., Toole T.E.O. (2011). Identification of Hypercoagulability in Dogs with Primary Immune-Mediated Hemolytic Anemia by Means of Thromboelastography. J. Am. Vet. Med. Assoc..

[66] Brooks A.C., Guillaumin J., Cooper E.S., Couto C.G. (2014). Effects of Hematocrit and Red Blood Cell-Independent Viscosity on Canine Thromboelastographic Tracings. Transfusion.

[67] Blois S.L., Côté E., Ettinger S., Feldman E. (2024). Chapter 171: Hyper- and Hypocoagulable States. Ettinger’s Textbook of Veterinary Internal Medicine.

[68] DIC and Thrombosis Cornell University College of Veterinary Medicine. https://www.vet.cornell.edu/animal-health-diagnostic-center/laboratories/comparative-coagulation/clinical-topics/dic-and-thrombosis?utm_source=chatgpt.com.

[69] Wiinberg B., Jensen A.L., Johansson P.I., Rozanski E., Tranholm M., Kristensen A.T. (2008). Thromboelastographic Evaluation of Hemostatic Function in Dogs with Disseminated Intravascular Coagulation. J. Vet. Intern. Med..

[70] Asakura H. (2016). Classifying Types of Disseminated Intravascular Coagulation: Clinical and Animal Models. Rinsho Ketsueki.

[71] Kittrell D., Berkwitt L. (2012). Hypercoagulability in Dogs Pathophysiology. Compend. Contin. Educ. Vet..

[72] Laurenson M.P., Hopper K., Herrera M.A., Johnson E.G. (2010). Concurrent Diseases and Conditions in Dogs with Splenic Vein Thrombosis. J. Vet. Intern. Med..

[73] Garcia-Sancho M., Saiz A., Rodriguez-Franco F., Villaescusa A., Rodriguez-Bertos A. (2010). Pulmonary Thromboembolism in a Dog with Inflammatory Bowel Disease. Rev. Complut. Cienc. Vet..

[74] Jacinto A.M.L., Ridyard A.E., Aroch I., Watson P.J., Morrison L.R., Chandler M.L., Kuzi S. (2017). Thromboembolism in Dogs with Protein-Losing Enteropathy with Non-Neoplastic Chronic Small Intestinal Disease. J. Am. Anim. Hosp. Assoc..

[75] Littman M.P., Dambach D.M., Vaden S.L., Giger U. (2000). Familial Protein-Losing Enteropathy and Protein-Losing Nephropathy in Soft Coated Wheaten Terriers: 222 Cases (1983–1997). J. Vet. Intern. Med..

[76] Sakamoto Y., Ishigaki K., Ishikawa C., Nakayama T., Asano K., Sakai M. (2020). Successful Management of Portal Vein Thrombosis in a Yorkshire Terrier with Protein-Losing Enteropathy. BMC Vet. Res..

[77] Wiinberg B., Kristensen A.T. (2010). Thromboelastography in Veterinary Medicine. Semin. Thromb. Hemost..

[78] Respess M., O’Toole T.E., Taeymans O., Rogers C.L., Johnston A., Webster C.R.L. (2012). Portal Vein Thrombosis in 33 Dogs: 1998–2011. J. Vet. Intern. Med..

[79] Kittrell D., Berkwitt L. (2012). Hypercoagulability in Dogs: Treatment. Compend. Contin. Educ. Vet..

[80] Hindmarsh D.D., Rutter C.R., Pugnetti V.D., Jeffery U. (2022). Response of the VCMVet Viscoelastic Coagulation Monitor to Veterinary Environmental Simulation Challenges. Vet. Clin. Pathol..

[81] Goggs R., Brainard B., De Laforcade A.M., Flatland B., Hanel R., Mcmichael M., Wiinberg B. (2014). Partnership on Rotational ViscoElastic Test Standardization (PROVETS): Evidence-Based Guidelines on Rotational Viscoelastic Assays in Veterinary Medicine. J. Vet. Emerg. Crit. Care.

